# Handgrip strength as an indicator of psychological status in hospitalized intensive care unit patients: a cross-sectional study

**DOI:** 10.3389/fmed.2026.1785328

**Published:** 2026-03-13

**Authors:** Zihang Chen, Yue He, Jing Wang, MinYing Zheng, Runtian Cao, Wenfeng Wen, Huiling Liang, Chunxi Lin, Wulamiding Kaisaier

**Affiliations:** 1 Department of Cardiology, The First Affiliated Hospital of Sun Yat-sen University, Guangzhou, China; 2 NHC Key Laboratory of Assisted Circulation and Vascular Diseases, Sun Yat-sen University, Guangzhou, China; 3 ISA Science City International School, Guangzhou, Guangdong, China; 4 Department of Neurology, The First Affiliated Hospital of Sun Yat-sen University, Guangzhou, Guangdong, China

**Keywords:** anxiety, cross-sectional study, depression, handgrip strength, intensive care unit (ICU)

## Abstract

**Background:**

Anxiety and depression are common among intensive care unit (ICU) patients, yet routine bedside assessment can be challenging. Whether handgrip strength can serve as a practical indicator of anxiety and depression in the ICU remains unclear.

**Methods:**

We consecutively enrolled patients admitted to the cardiac intensive care unit (CICU) of a large public hospital in South China between July 2022 and October 2023. Handgrip strength was measured using a calibrated dynamometer, and anxiety and depression were assessed using the Hospital Anxiety and Depression Scale (HADS). Logistic regression was used to examine associations between handgrip strength and anxiety/depression adjusting for relevant covariates.

**Results:**

A total of 308 CICU inpatients were ultimately included in this cross-sectional analysis. The median age was 60 years (IQR: 50–69), and mean handgrip strength was 29.5 ± 11.3 kg. In multivariate logistic regression, lower handgrip strength was independently associated with anxiety (OR: 0.94 [95% CI: 0.90–0.98]) and depression (OR: 0.94 [95% CI: 0.90–0.99]). In addition, administration of sedatives was associated with high odds of anxiety (OR: 2.24 [95% CI: 1.16–4.31]).

**Strengths and limitations:**

Multivariate logistic regression was used to adjust for potential confounding factors. As a single-center cross-sectional study, selection and center-specific biases were unavoidable; further details are provided in the Limitations and Future Directions section.

**Conclusions:**

Handgrip strength is a non-invasive, readily accessible measure that may help identify patients at risk of anxiety and depression in the ICU. Potential mechanisms include impaired self-efficacy and inflammatory activation. Future studies should validate predictive thresholds and clarify temporal and mechanistic relationships.

## Highlights

- To evaluate handgrip strength as a simple bedside indicator of anxiety and depression in ICU patients.
- To highlight the interaction between physical function and psychological status in critically ill cardiovascular patients.
- To explore whether the association between handgrip strength and psychological symptoms varies by sleep quality, age, and sex.

## Introduction

Given the declining mortality of critically ill patients requiring intensive care unit (ICU) admission, interest has increased in understanding the psychological sequelae associated with intensive care hospitalization over the past decade (1). Within the critical care milieu, traumatic memories of emergency procedures and persistent sleep disturbance may exacerbate psychological comorbidities (2, 3). Post-Intensive Care Syndrome (PICS), a constellation of persistent physical, cognitive, and psychiatric impairments—now constitutes a critical clinical challenge among ICU survivors (4). Anxiety and depression are highly prevalent in this population, with prevalence exceeding 50% in longitudinal cohorts (5–7), and are associated with poor quality of life and higher post-discharge mortality (3, 8).

Psychological assessment tools used in the ICU are often impractical, driving the need for a simple alternative indicator. The Hospital Anxiety and Depression Scale (HADS) was developed to measure the anxiety and depression of patients during hospitalization, also considered reliable in critical care settings (9, 10). However, its performance can be affected by translation and cross-cultural differences (11). Therefore, it is significant to identify valid indicators for psychological assessment. Handgrip strength, a valid measure of muscular function, is inversely associated with anxiety and depression across multiple populations (12–15), and therefore suggests its potential to serve as an indicator.

The value of handgrip strength for evaluating psychological status in ICU patients remains unclear, prompting the present study. The aim of our research is to explore whether handgrip strength could serve as an indicator for anxiety and depression of patients in ICU, and the other possible impact factors.

## Methods

### Procedure

We consecutively recruited patients admitted to the CICU of a large tertiary hospital in South China, including direct admission via the emergency department and transfer from other departments, between July 1, 2022, and October 31, 2023.

Data were collected using a standardized protocol covering three domains. Trained researchers collected demographic and psychometric data, such as participants' gender, age, smoking status, Pittsburgh Sleep Quality Index (PSQI) scores, and Hospital Anxiety and Depression Scale (HADS) metrics via structured interviews at 72 h after admission. Concurrently, clinical parameters including principal admission diagnoses, the usage of sedative drugs and essential laboratory biomarkers were retrieved from the institutional electronic health records system.

The inclusion criteria encompassed three principal components: (1) Age ≥ 16 years at enrollment; (2) Minimum CICU hospitalization duration of 72 h; (3) Neurologically intact individuals capable of independent questionnaire completion or nurse-assisted verbal responses. Exclusion criteria were systematically defined as: (1) Severe psychological issues or critical condition; (2) Impaired consciousness or inability to cooperate with researchers to complete the assessments; (3) Sensory or communication barriers including uncorrected visual/hearing deficits or clinically significant aphasia.

**Figure 1:**
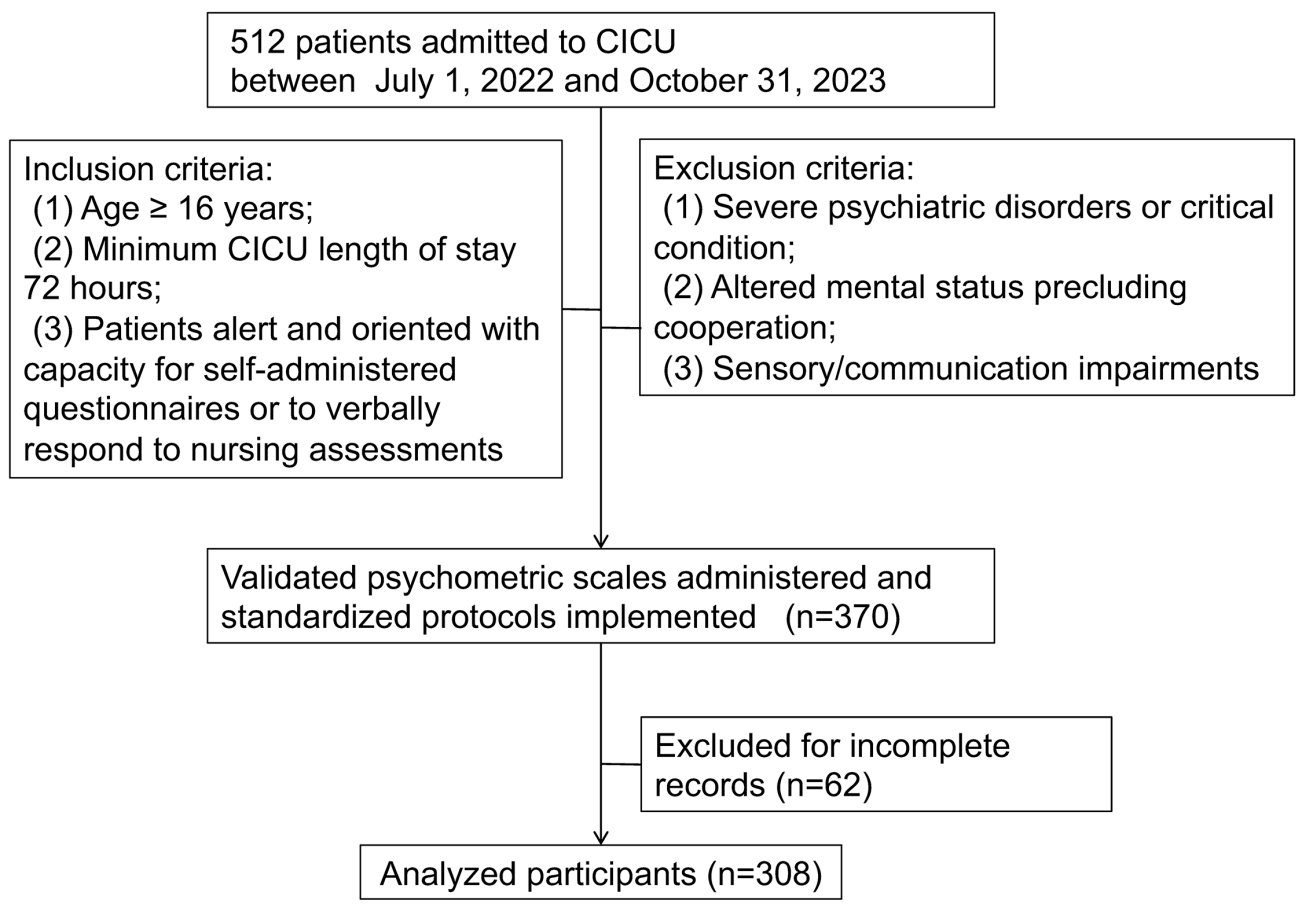
Patient selection flowchart. CICU, Cardiac Intensive Care Unit.

A total of 308 CICU inpatients meeting the predefined inclusion criteria were ultimately enrolled in the analysis (Figure 1). The study population exhibited a median age of 60 (IQR: 50–69) years, with a male predominance (66.9%, *n* = 206).

### Measures

Diagnostic categorization followed the International Classification of Diseases, 10th Revision (ICD-10) criteria. Notably, cardiovascular diagnoses were stratified into three distinct categories: acute coronary syndrome [acute ST segment elevation myocardial infarction (ICD-10 code I21.300), acute non-ST segment elevation myocardial infarction (ICD-10 code I21.400), and unstable angina (I20)], heart failure (I50), and other diagnoses. This tripartite classification framework facilitated subsequent comparative analyses across diagnostic subgroups.

Handgrip strength was measured according to a standardized dynamometry protocol. Measurements were performed 72 h post-CICU admission using a calibrated SUNSSUN-BM101 dynamometer (measurement range: 5–100 kg, resolution: 0.1 kg). Following established biomechanical guidelines, patients were tested in a seated position (or supine if non-ambulant) with the elbow flexed at 90°, forearm and wrist in neutral position (16, 17). We performed duplicate bilateral measurements with a 60 s rest between trials to minimize fatigue. The peak value across trials was documented for subsequent analysis.

Sedative administration in this investigation encompassed all pharmacological interventions initiated upon CICU admission, specifically γ-aminobutyric acid (GABA) -ergic agents administered per standardized CICU protocols. The therapeutic regimen included benzodiazepine derivatives such as midazolam, alprazolam, and non-benzodiazepine hypnotics such as zolpidem tartrate.

The Pittsburgh Sleep Quality Index (PSQI) was systematically employed to quantify sleep quality during the preceding 30-day observational period. This validated instrument encompasses seven critical sleep parameters: perceived sleep quality, latency to sleep onset, total sleep duration, sleep efficiency quotient, frequency of nocturnal disturbances, pharmacologic sleep aid utilization, and diurnal functional impairment. Each item is scored on a 4-point Likert scale (0–3), with a total score ranging from 0 to 21. Notably, elevated scores demonstrated a significant inverse correlation with sleep integrity metrics, where increasing values correspond to progressively diminished sleep quality (18).

The Hospital Anxiety and Depression Scale (HADS) incorporates a 14-item psychometric instrument, systematically bifurcated into two discrete subscales: seven items evaluating anxiety symptomatology (HADS-A) and seven parallel items assessing depressive manifestations (HADS-D). Each subscale employs a four-tiered Likert scaling system 0–3, consequently generating cumulative domain-specific scores spanning 0–21. Notably, ascending composite values demonstrate a proportional association with amplified clinical severity of the respective affective disorders (19). In accordance with established psychometric protocols, participants were dichotomized using a cutoff score of 8 for subsequent analyses (6). Hence, HADS-A and HADS-D scores were both dichotomized into binary outcome groups.

### Statistical analysis

All statistical analyses were performed using R software (version 4.2.2). Normally distributed continuous data were presented as mean ± standard deviation, and non-normal data as median (interquartile range). Student's *t*-test or Mann-Whitney U test was used accordingly. Normality was checked using Shapiro-Wilk test and Q-Q plots. Categorical variables were presented as *n* (%) and compared using χ^2^ or Fisher's exact test when cell counts were <5.

A selection of candidate variables (*P* < 0.1) was filtered out through univariate analyses, encompassing age, sex, PSQI scores, handgrip strength, and sedative use; these variables were further included in the multivariate analysis for in-depth assessment. In parallel, to control for confounding biases, several clinically important variables for critically ill cardiovascular patients—including BMI, New York Heart Association (NYHA) functional classification, and serum albumin levels—were also integrated into the multivariate regression models. We also computed the variance inflation factor (VIF) for each variable to assess multicollinearity. To address incomplete observations, missing data patterns underwent multiple imputation using chained equations (MICE) with 20 imputed datasets and 5 iteration cycles. This method preserved covariance structure and reduced imputation bias, with convergence confirmed by diagnostic checks.

In our sensitivity analysis, we re-analyzed HADS-A and HADS-D scores as continuous outcomes to assess the robustness of the primary findings. Linear regression was used, with Model 1 unadjusted and Model 2 adjusted for age, sex, and serum albumin (ALB). Subgroup analyses were systematically executed in a sequential manner to identify the specific conditions under which the correlation was more pronounced. Initially, subgroup analyses were conducted following the multivariate regression outcomes to elucidate potential effect modifications and identify subgroups exhibiting heightened associations. Subsequently, to comprehensively delineate the relationship between handgrip strength and total HADS scores treated as continuous variables, Pearson correlation coefficients were computed for the overall cohort and stratified subgroups. Notably, all statistical inferences were derived using a predefined significance criterion of two-tailed *p* < 0.05.

## Results

### Basic characteristics

Baseline demographic and clinical characteristics are comprehensively summarized in Table 1. Mean handgrip strength across participants was quantified as 29.5 ± 11.3 kg. Stratified by the 8-point threshold, notably, the HADS-A outcome group demonstrated statistically significant intergroup disparities in sex distribution, PSQI scores, sedative use, and handgrip strength (*p* < 0.05). Conversely, within the HADS-D outcome cohort, marked differences were observed in age, PSQI scores, and handgrip strength (*p* < 0.05) (Table 1).

**Table 1:** Baseline characteristics.

**Factor**	**Overall**	**HADS-A score**		***P***	**HADS-D score**		***P***
		**<8**	≥**8**		**<8**	≥**8**	
Population (*n*)	308	251	57		261	47	
Age (y)	60.0 (50.0, 69.0)	60.0 (50.0, 67.0)	61.0 (53.0, 73.0)	0.137	59.0 (49.0, 68.0)	61.0 (57.0, 71.5)	0.034
**Sex**, ***n*** **(%)**							
Male	206 (66.9)	175 (69.7)	31 (54.4)	0.039	177 (67.8)	29 (61.7)	0.515
Female	102 (33.1)	76 (30.3)	26 (45.6)		84 (32.2)	18 (38.3)	
**Smoking**, ***n*** **(%)**							
Yes	185 (60.1)	152 (60.6)	33 (57.9)	0.825	158 (60.5)	27 (57.5)	0.813
No	123 (39.9)	99 (39.4)	24 (42.1)		103 (39.5)	20 (42.6)	
**CVDs**, ***n*** **(%)**							
No CVDs	161 (52.27)	133 (52.99)	28 (49.12)	0.6891	134 (51.34)	27 (57.45)	0.6917
ACS	94 (30.52)	77 (30.68)	17 (29.82)		82 (31.42)	12 (25.53)	
CCS	53 (17.21)	41 (16.33)	12 (21.05)		45 (17.24)	8 (17.02)	
**ACS**							
No	214 (69.48)	174 (69.32)	40 (70.18)	1	179 (68.58)	35 (74.47)	0.5257
Yes	94 (30.52)	77 (30.68)	17 (29.82)		82 (31.42)	12 (25.53)	
**NYHA**, ***n*** **(%)**							
I/II	267 (86.69)	219 (87.25)	48 (84.21)	0.6935	228 (87.36)	39 (82.98)	0.5619
III/IV	41 (13.31)	32 (12.75)	9 (15.79)		33 (12.64)	8 (17.02)	
BMI (kg/m^2^)	24.0 (22.0, 27.0)	24.3 (22.0, 27.0)	23.0 (21.0, 26.4)	0.086	24.3 (22.0, 27.0)	23.0 (21.0, 26.0)	0.0807
SBP (mmHg)	122 (112, 135)	122 (112, 136)	121 (110, 134)	0.721	122 (112,134)	125 (110, 137)	0.966
DBP (mmHg)	74 (67, 82)	74 (67,82)	72 (65, 80)	0.186	74 (67, 82)	74 (67, 82)	0.485
Serum creatinine (umol/L)	78.0 (65.8, 95.0)	79.0 (66.0, 95.0)	74.0 (64.0, 102.0)	0.698	78.0 (66.0, 95.0)	76.0 (65.0, 99.0)	0.701
Serum albumin (g/L)	40.0 (37.0, 43.0)	41.0 (37.6, 43.0)	40.0 (37.0, 43.0)	0.385	40.0 (38.0, 43.0)	39.0 (36.2, 44.0)	0.398
NT-proBNP (pg/ml)	287.5 (57.6, 1155.5)	297.0 (57.3, 1126.0)	211.0 (58.3, 1193.0)	0.851	253.0 (54.1, 1124.0)	434.0 (66.6, 1654.5)	0.273
PSQI score, (point)	6.0 (4.0, 9.0)	6.0 (4.0, 9.0)	8.0 (5.0, 11.0)	0.012	6.0 (4.0, 9.0)	8.0 (4.5,11.5)	0.026
Handgrip strength (kg), mean (SD)	29.5 (11.3)	30.8 (11.0)	23.9 (10.9)	<0.0001	30.4 (11.0)	24.2 (11.4)	0.0005
**Sedative usage**, ***n*** **(%)**							
No	236 (76.62)	201 (80.08)	35 (61.40)	0.0046	205 (78.54)	31 (65.96)	0.0911
Yes	72 (23.38)	50 (19.92)	22 (38.60)		56 (21.46)	16 (34.04)	

Values are presented as median (interquartile range) unless otherwise indicated. HADS-A, The Hospital Anxiety and Depression Scale for anxiety; HADS-D, The Hospital Anxiety and Depression Scale for depression; BMI, body mass index; SBP, Systolic blood pressure; DBP, Diastolic blood pressure; NT-proBNP, N-terminal pro-B-type natriuretic peptide; PSQI, Pittsburgh Sleep Quality Index.

### Main findings

**Figure 2:**
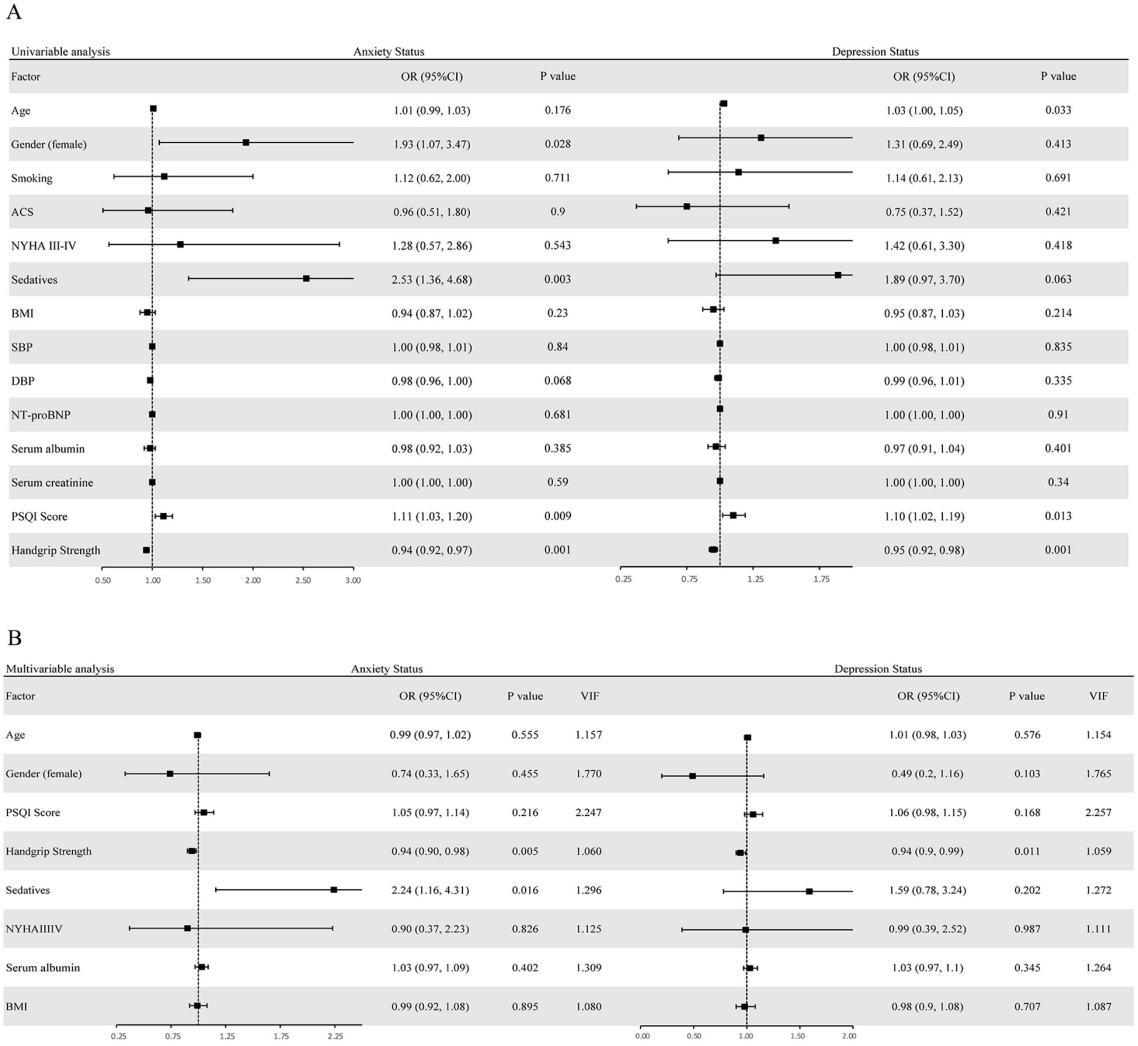
Univariate **(A)** and multivariate **(B)** logistic analyses of HADS-A and HADS-D scores, including odds ratios (OR) and variance inflation factors (VIF).

Univariate logistic regression analyses delineated distinct predictive factors for anxiety and depression in the study cohort. Female sex (OR: 1.93, 95% CI: 1.07–3.47), elevated PSQI scores (OR: 1.11, 95% CI: 1.03–1.20), administration of sedatives (OR: 2.53, 95% CI: 1.36–4.68), and reduced handgrip strength (OR: 0.94, 95% CI: 0.92–0.97) were significantly associated with higher HADS-A scores, reflecting a greater likelihood of anxiety. In parallel, advanced age (OR: 1.03, 95% CI: 1.00–1.05), higher PSQI scores (OR: 1.10, 95% CI: 1.02–1.19), and decreased handgrip strength (OR: 0.95, 95% CI: 0.92–0.98) correlated observably with elevated HADS-D scores, signifying an elevated risk of depression (Figure 2A).

Multivariate logistic regression showed that lower handgrip strength was an independent predictor of higher HADS-A (OR: 0.94, 95% CI: 0.90–0.98) and HADS-D (OR: 0.94, 95% CI: 0.90–0.99) scores. Specifically, each 1 kg increase in handgrip strength was associated with a 6% lower risk of anxiety and depression, respectively. Additionally, administration of sedatives retained a significant association with increased HADS-A scores (OR: 2.24, 95% CI: 1.16–4.31) following adjustment for all covariates, highlighting its independent role in anxiety risk. Furthermore, the values of VIF for all variables were less than 3, indicating that multicollinearity was negligible, and no further adjustment of variables was required (Figure 2B).

### Sensitivity analysis

In the crude model, higher handgrip strength was associated with lower anxiety (HADS-A) scores (β = −0.0542; 95% CI, −0.0920 to −0.0163; *P* = 0.005), and this remained significant after adjustment (β = −0.0602; 95% CI, −0.1154 to −0.0050; *P* = 0.033). Similarly, maximal handgrip strength was inversely associated with depressive symptoms in the crude model (β = −0.0786; 95% CI, −0.1151 to −0.0420; *P* = 3.07 × 10^−5^) and adjusted model (β = −0.0919; 95% CI, −0.1453 to −0.0386; *P* = 7.87 × 10^−4^). Overall, the inverse associations between handgrip strength and anxiety/depression were consistent across models, supporting the robustness of the results (Table 2).

**Table 2:** Linear regression analyses of maximal handgrip strength in relation to anxiety (HADS-A) and depression (HADS-D) scores: sensitivity analysis.

**Outcome**	**Model 1 (crude)**	***P* value**	**Model 2 (adjusted)**	***P* value**
HADS-A	−0.0542 (−0.0920, −0.0163)	0.005	−0.0602 (−0.1154, −0.0050)	0.033
HADS-D	−0.0786 (−0.1151, −0.0420)	<0.001	−0.0919 (−0.1453, −0.0386)	0.001

### Subgroup analysis

**Figure 3:**
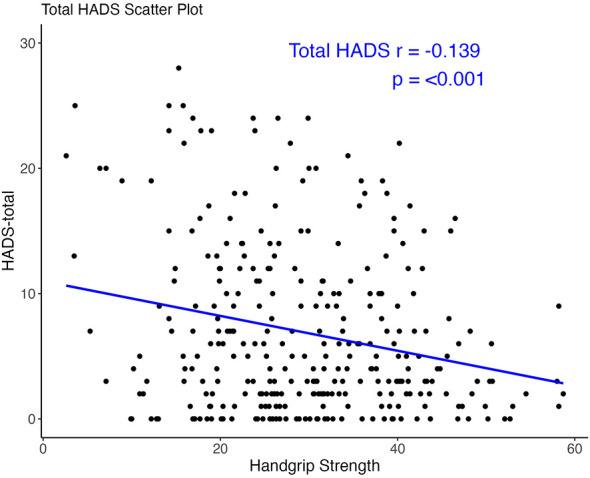
Linear relationship between handgrip strength and psychological status.

**Figure 4:**
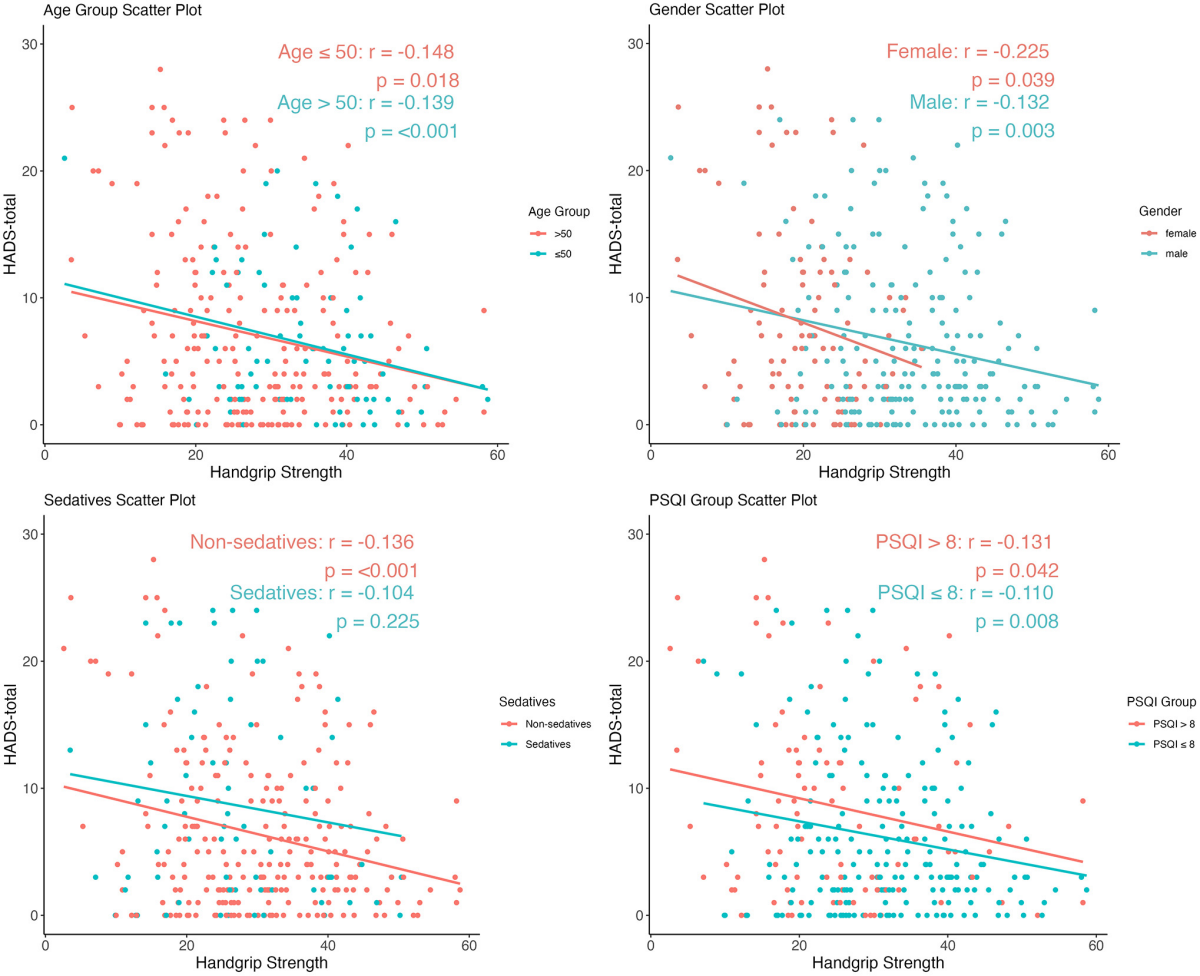
Linear relationships between handgrip strength and psychological status stratified by age, sex, sedative use, and PSQI score.

Notably, the bivariate correlation analysis demonstrated a significant inverse association between handgrip strength and total HADS scores in the entire cohort (*r* = −0.139, *p* < 0.001) (Figure 3). This relationship persisted consistently across all predefined subgroups: age-stratified populations (for age ≤ 50, *r* = −0.148, *p* = 0.018; for age > 50, *r* = −0.139, *p* < 0.001), sex-based partitions (for female, *r* = −0.225, *p* = 0.039; for male, *r* = −0.132, *p* = 0.003), sedative use status (for no sedative use, *r* = −0.136, *p* < 0.001; for sedative use, *r* = −0.104, *p* = 0.225), and sleep quality strata (for PSQI score > 8, *r* = −0.131, *p* = 0.042; for PSQI score ≤ 8, *r* = −0.110, *p* = 0.008) (Figure 4).

## Discussion

### Comparison with previous studies

Handgrip strength may serve as a simple, non-invasive indicator of poor psychological status. Growing epidemiological evidence supports an inverse association between handgrip strength and poor psychological status, including anxiety (20) and depression (21–23), across diverse populations (12, 24). Our multivariate logistic regression confirmed this inverse relationship and extended it to critically ill patients in the ICU.

### Clinical implications

Compared with the HADS scale, handgrip strength measurement is simple and time-saving, supporting its use as a non-invasive indicator of inverse psychological status. Early identification of high-risk patients using handgrip strength may allow earlier psychological intervention, which can improve prognosis and reduce mortality in ICU patients (25).

### Potential mechanisms

Reduced self-efficacy related to low handgrip strength may explain this inverse association. Handgrip strength constitutes a fundamental metric for evaluating muscle function and general physical performance (26). Poor physical function may impair self-efficacy and promote negative cognitive patterns, increasing anxiety, and depression (27, 28). These findings highlight the close interplay between physical and psychological health. In addition, elevated circulating pro-inflammatory cytokines exhibit bidirectional relationships with both diminished muscular capacity and psychological symptom emergence (29, 30), suggesting that inflammation may mediate the relationship between low handgrip strength and poor psychological status.

Sleep quality is another key determinant of psychological status, with neurobiological pathways linking sleep to physical function. Substantial evidence demonstrates that diminished sleep quality is positively associated with exacerbated anxiety and depression severity in heterogeneous patient cohorts (31, 32). This point is substantiated by the univariate logistic regression analyses of our study, and consistent with the stronger correlation between handgrip strength and poor psychological status in patients with poorer sleep quality. However, this association was no longer significant in multivariate analysis, possibly because patients with poor sleep quality have worse physical status, which is reflected in reduced handgrip strength, and this further induces poor psychological conditions. Studies have confirmed that sleep-wake disorders are correlated with low handgrip strength (33). Poor sleep quality induces reduced protein synthesis and/or enhanced protein catabolism, thereby decreasing muscle mass and further impairing handgrip strength (34).

The higher risk of poor psychological status in female and elderly patients may also be mediated by reduced physical function. Sex modulates psychological vulnerability profiles, with data confirming higher anxiety prevalence and increased depression risk in females vs. males (27, 35, 36). Our study substantiates these epidemiological patterns in univariate logistic regression analyses. Different psychological disorders exhibit distinct age distribution patterns. In males, the incidence of depression shows a linear increase with advancing age, whereas this trend is more complex in female patients (37). As for anxiety, multiple peaks emerge across different age stages, presenting a fluctuating pattern (38). The positive correlation between male and anxiety observed in our study may be attributed to the relatively high proportion of male patients included. Parallel to sleep quality, the correlation from gender and age to psychological status becomes insignificant in multivariate regression analyses, indicating reduced handgrip strength may act as a mediator. In aging, handgrip strength gradually decreases after the peak, and the females exhibit weaker handgrip strength than the male (39). Corresponding to this, skeletal muscle mass decreases in the older patients (40) and men have a higher ratio of muscle to fat, contrary to women (41). Our hypothesis is supported by these aforementioned findings.

In the ICU, sedatives are more frequently administered to patients with high levels of anxiety. The appropriate use of analgesic and sedative medications to maintain an optimal level of pain control and sedation is recommended in the ICU setting, as it helps patients cope with pain, enhances comfort levels and shortens hospital stays (42, 43). Evidence has demonstrated that the combined use of sedatives and analgesics may ameliorate the detrimental stress response in ICU patients (44). Our study findings indicate that patients receiving sedative medications exhibit higher anxiety levels. Attributing this finding to a direct anxiogenic effect of sedatives would contradict previous studies. Given the cross-sectional design, a more plausible interpretation is that ICU clinicians are more inclined to prescribe sedatives to patients with elevated anxiety levels. To further clarify the relationship between these two factors, a feasible approach would be to extend the observation period for each patient and compare the impact of sedative use based on changes in their anxiety levels over time.

### Strengths

To enhance the representativeness of the study population, we used consecutive sampling of CICU admissions within the study period. Standardized protocols were applied for HADS administration and handgrip dynamometry to reduce measurement variability, and clinically relevant covariates were incorporated into multivariate models.

### Limitations and future directions

The study design introduced unavoidable limitations and potential biases. This study was designed as a single-center cross-sectional study, so it is difficult to distinguish the temporal sequence of variables and control for confounding factors. Moreover, this study is susceptible to selection bias and center-specific bias, which limits the extrapolation of its findings (45, 46). Longitudinal models and multi-center studies should be designed to address the current issues in the future.

We did not fully account for all potential confounders during patient enrollment. Participants with missing outcome variables were excluded during sample enrollment. This may introduce missing-data bias, reduce sample size, and lower statistical power. When enrolling the study sample, we did not take into account whether the patients had neuromuscular or musculoskeletal diseases, or a history of hand-related surgery. Previous studies have indicated that patients with such conditions, including rheumatic diseases and osteoarthritis, tend to have reduced handgrip strength (47, 48), and other studies have suggested that these patients face a significantly higher risk of developing anxiety and depression (49, 50). The decline in handgrip strength and poor psychological health status caused by these chronic conditions most likely existed prior to hospital admission, thereby interfering with the interpretation of our study findings.

We did not collect comprehensive data on factors related to both handgrip strength and psychological status. We failed to consider the patients' medication history before admission. Studies have shown that the use of certain medications such as nitroglycerin (51), ticagrelor (52), and opioids (53) may increase the risk of anxiety or depression, whereas the administration of aspirin (54) and statins (55, 56) could reduce the risk of depression. This factor has also confounded the analysis of the patients' psychological health status. Pain is highly prevalent among ICU patients (57), but we did not conduct an investigation on the presence of arm pain in the patients, yet such pain can exert an impact on both handgrip strength (58) and psychological status (59), which constitutes another important confounding factor that may affect the interpretation of the study results. Besides sedatives, the administration of analgesics is also a key aspect of ICU clinical practice (60). In the present study, we did not analyze the types, dosages, administration regimens, and duration of use of these agents. Therefore, more detailed recording and analysis of such data should be conducted in future research to explore the impact of different sedative and analgesic regimens on patients' psychological health.

A quantitative relationship between handgrip strength and psychological status was not established. A cutoff value of ≥8 was used to categorize the severity of anxiety and depression. This approach aligns with the requirement for rapid and convenient assessment in the ICU setting but compromises the statistical power. Our findings only demonstrate that handgrip strength can serve as a potential indicator for anxiety and depression; however, the specific correlation between the numerical values of handgrip strength and the severity of anxiety/depression remains unclear. This issue warrants further investigation in future research.

## Conclusions

Handgrip strength is inversely associated with poor psychological status in ICU patients, possibly mediated by reduced self-efficacy. This association underscores the close link between physical and psychological health. Sleep quality, age, and sex are also associated with poor psychological status, and handgrip strength may act as a mediator. The relationship between sedative use and psychological status requires further clarification. Future studies should establish quantitative diagnostic thresholds and explore the underlying mechanisms in greater depth. Overall, routine bedside measurement of handgrip strength is recommended for the early screening of anxiety and depression in ICU patients.

## Data Availability

The original contributions presented in the study are included in the article/supplementary material, further inquiries can be directed to the corresponding authors.
